# Anxiety-like Behavior and GABAAR/BDZ Binding Site Response to Progesterone Withdrawal in a Stress-Vulnerable Strain, the Wistar Kyoto Rats

**DOI:** 10.3390/ijms23137259

**Published:** 2022-06-30

**Authors:** Dannia Islas-Preciado, Gabriela Ugalde-Fuentes, Isabel Sollozo-Dupont, María Eva González Trujano, Nancy Cervantes-Anaya, Erika Estrada-Camarena, Carolina López-Rubalcava

**Affiliations:** 1 Lab de Neuropsicofarmacología, Dirección de Neurociencias, Instituto Nacional de Psiquiatría Ramón de la Fuente Muñiz, Ciudad de México 14370, Mexico; dislas@imp.edu.mx (D.I.-P.); ugalde_gaby@hotmail.com (G.U.-F.); sodi8507@gmail.com (I.S.-D.); qfb.nancy.cervantes@hotmail.com (N.C.-A.); 2 Lab de Neurofarmacología de Productos Naturales, Dirección de Neurociencias, Instituto Nacional de Psiquiatría Ramón de la Fuente Muñiz, Ciudad de México 14370, Mexico; evag@imp.edu.mx; 3 Departamento de Farmacobiología, Centro de Investigación y de Estudios Avanzados IPN (Cinvestav-IPN), Ciudad de México 07360, Mexico

**Keywords:** anxiety-like behavior, progesterone withdrawal, BDZ site in the GABAA receptor, HPA axis function

## Abstract

Stress susceptibility could play a role in developing premenstrual anxiety due to abnormalities in the hypothalamus–pituitary–adrenal (HPA) axis and impairments in the GABAA receptors’ benzodiazepine (BDZ) site. Hence, we studied the stress-vulnerable Wistar Kyoto rat strain (WKY) to evaluate progesterone withdrawal (PW) effects on anxiety, HPA axis response, and to explore indicators of GABAA functionality in the BDZ site. For five days, ovariectomized WKY rats were administered 2.0 mg/kg of progesterone. Twenty-four hours after the last administration, rats were tested in the anxiety-like burying behavior test (BBT) or elevated plus maze test (EPM), and corticosterone was determined. [3H]Flunitrazepam binding autoradiography served as the BDZ binding site index of the GABAA receptor in amygdala nuclei and hippocampus’s dentate gyrus (DG). Finally, different doses of diazepam in PW-WKY rats were tested in the BBT. PW induced anxiety-like behaviors in both BBT and EPM compared with No-PW rats. PW increased corticosterone, but was blunted when combined with PW and BBT. PW increased [3H]Flunitrazepam binding in the DG and central amygdala compared with No-PW rats. Diazepam at a low dose induced an anxiogenic-like response in PW rats, suggesting a paradoxical response to benzodiazepines. Overall, PW induced anxiety-like behavior, a blunted HPA axis response, and higher GABAAR/BZD binding site sensitivity in a stress-vulnerable rat strain. These findings demonstrate the role of stress-susceptibility in GABAAR functionality in a preclinical approximation of PMDD.

## 1. Introduction

Premenstrual dysphoric disorder (PMDD) is the severe form of premenstrual syndrome [1,2] characterized by the recurrence of negative mood symptoms such as irritability, anxiety, and depression, disrupting the daily life of suffering women [1]. Clinical evidence indicates that anxiety is one of the most common emotional symptoms reported in PMDD patients [3,4,5]. This anxiety could be related to the drop in progesterone and allopregnanolone levels during the late luteal phase of the menstrual cycle [6,7,8].

Moreover, the hypothalamus–pituitary–adrenal (HPA) axis seems to be involved in the etiology of PMDD symptoms [9,10,11]. The HPA axis activation initiates with the synthesis of corticotropin-releasing hormone (CRH) in the paraventricular nucleus, and reaches the pituitary gland to produce adrenocorticotropic hormone (ACTH). ACTH integrates into the blood circulation and interacts with receptors of the adrenal cortex to synthesize cortisol, the main product of HPA axis activation [12,13]. Cortisol reaches glucocorticoid or mineralocorticoid receptors in the hypothalamus, hippocampus or amygdala, among other brain structures [14]. Under chronic stress or after severe stress, the HPA axis might be dysregulated due to changes in the sensitivity of glucocorticoid receptors due to continuous hypercortisolism exposure [15].

Previous studies in women have shown a blunted response of the adrenocorticotropic hormone in the luteal phase [16] and cortisol in response to stress, compared with control women in both follicular [9,17] and luteal phase [9,17,18]. Additionally, the cortisol awakening response is flattened in women with premenstrual disorders in relation to healthy controls without premenstrual disorders [19,20]. The link between stress and premenstrual mood disorders is strong; for example, higher stress levels may trigger or exacerbate PMDD symptoms [21]. Even more, higher perceived stress was found in women with premenstrual symptoms [22], and a previous report found increased rates of premenstrual mood disorders in women who experienced posttraumatic stress symptoms [23]. Hence, stress susceptibility could play a relevant role in developing symptoms related to the premenstrual period. The literature suggests that stress promotes changes in the gamma-aminobutyric acid (GABA) receptor subunits’ composition affecting their sensitivity and functionality [24,25,26,27]. Reports showed changes in the reactivity of brain areas involved in PMDD emotional regulation. Thus, the main structures involved are cortico-limbic regions, since increased amygdala reactivity and decreased fronto-cortical activity were reported during negative emotions [28,29,30]. In turn, these changes were associated with progesterone levels [30]. Additionally, decreased GABA brain levels [31] and decreased sensitivity of GABAAR to allopregnanolone, a progesterone metabolite, were reported in women with PMDD [32]. Moreover, as GABAARs are widely expressed in the hippocampus and the amygdala [33], and these areas are involved in HPA axis regulation [15], it is expected that alterations in GABAAR functionality impact the HPA axis activity. 

Regarding preclinical models, Wistar Kyoto rats (WKY) are considered a stress-vulnerable strain [34,35,36] that show an exaggerated anxiety-like response when subjected to an experimental approximation for premenstrual anxiety such as the progesterone withdrawal (PW) challenge [37]. WKY rats have shown hyperactivity of the HPA axis in stressful conditions [38,39,40], and higher anxiety-like behaviors [41]. Interestingly, lower corticosterone levels were detected in male and female WKY rats after social isolation [42], and in males after chronic restraint stress [43]. These findings suggest that psycho-emotional or physical stress may lead to a blunted corticosterone response in WKY rats. Studies in women with premenstrual disorders showed that physical stress due to exercise [10] or psycho-emotional stress induced by a social stress test [44] induced a similar blunted HPA axis reactivity. The hippocampus and amygdala nuclei are brain structures strongly involved in fear and anxiety [45,46] as well as HPA axis regulation [12]. Studies have reported sub-regional differences in stress-integrative functions depending on the stressor type. Anisman and Matheson (2005) classified stressor types into “processive and systemic” stressors. In this background, the central amygdala is highly responsive to systemic stressors (such as inflammatory challenges). In contrast, medial and basolateral amygdala may be more reactive to processive stressors, such as noise or forced swimming [15,47]. Thus, differential modulation from the PW challenge and exposure to a behavioral test might be expected in the present study based on the brain area analyzed.

Therefore, to test whether WKY rats showed a blunted response in the HPA axis and altered sensitivity of GABAAR function in response to PW, we assessed the corticosterone plasmatic levels and the BDZ binding site in the GABAA receptor in response to PW. An additional experiment evaluated the pharmacological sensitivity to diazepam in female WKY rats subjected to PW. Diazepam is a reference benzodiazepine that exerts its anxiolytic effects through GABAAR in behavioral paradigms [48,49,50]. Moreover, the effects of diazepam may vary according to stress susceptibility [51] and have not been previously described in female WKY rats subjected to PW. We hypothesized that WKY rats would show differential GABAAR response due to stress-susceptibility by revealing changes in their sensitivity to diazepam, and high anxiety-like response to PW.

## 2. Results

### 2.1. Progesterone Withdrawal Induced Anxiety-like Behaviors

**Figure 1:**
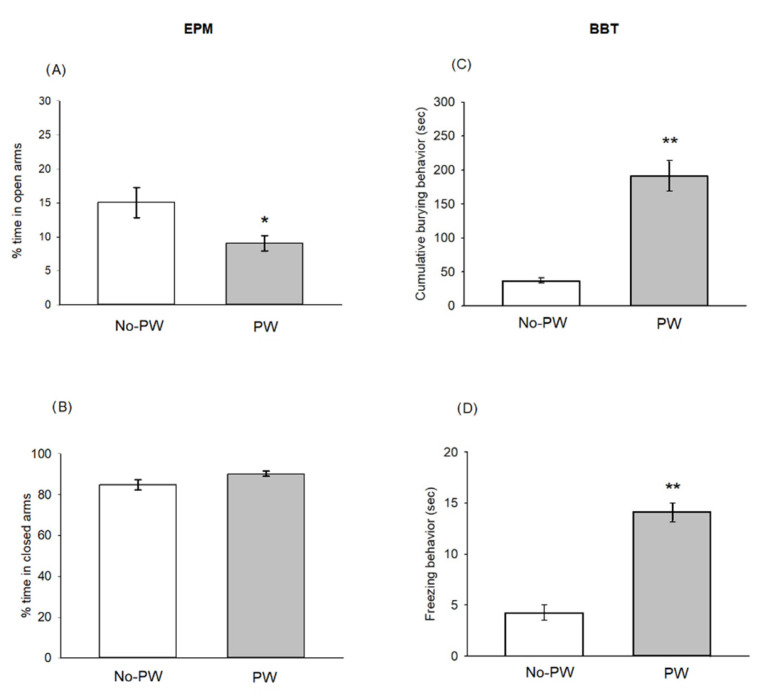
Left panels show the effects of progesterone withdrawal (PW) in Wistar Kyoto rats on the % of time spent in open arms (**A**), and the % of time spent in closed arms (**B**), in the elevated plus maze test (n = 6 per group). The right panels show the effects of PW on cumulative burying behavior (**C**), and freezing behavior (**D**), in the burying behavior test (n = 15 No-PW, n = 14 PW). Each bar represents the mean ± standard error of the mean. * *p* < 0.05, ** *p* < 0.01 vs. No-PW group.

As observed in Figure 1A, PW induced an anxiogenic-like effect by reducing the percentage of time spent in open arms in the EPM (t = 2.589, *p* < 0.05), but no significant changes were detected in the percentage of time in the closed arms compared with the No-PW group (t = 1.899, *p* = 0.08) (Figure 1B).

On the other hand, Figure 1C shows that PW increased cumulative burying behavior in the BBT when compared with the No-PW group (t = 6.965, *p* < 0.001). Similarly, it can be seen that PW increased freezing behavior when compared with No-PW animals (t = 8.137, *p* < 0.001) (Figure 1D).

### 2.2. Locomotor Activity Was Not Altered Due to PW or Diazepam Administration

The locomotor activity test did not show differences in the number of squares crossed during the 5 min session due to PW or diazepam treatment (Table 1). The total number of crosses registered in the EPM served as a locomotor activity index, and no significant changes were detected due to PW (not shown). Therefore, motor alterations that might interfere with the animal’s behavioral performance could be discarded.

### 2.3. Corticosterone Serum Levels

**Figure 2:**
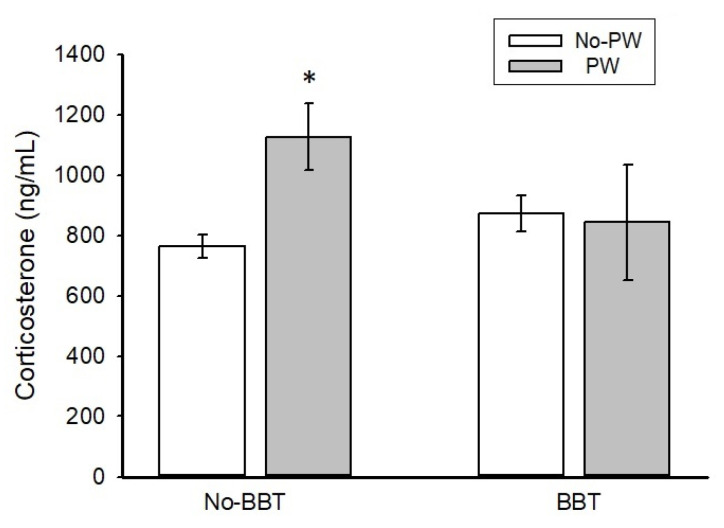
Corticosterone levels of Wistar Kyoto rats subjected to progesterone withdrawal. Each bar represents the mean ± standard error of the mean in ng/mL n = 4–6 per group. * *p* < 0.05 versus No-PW = no progesterone withdrawal (vehicle-treated rats); PW = progesterone withdrawal.

Figure 2 shows corticosterone concentrations in ng/mL. The BBT exposure did not alter corticosterone values in No-PW rats. On the other hand, PW increased corticosterone only in No-BBT rats (*p* < 0.01) when compared with No-PW animals (*p* < 0.01). Two-way ANOVA values were obtained for treatment F1,18 = 2.844, *p* < 0.05, behavioral test F1,18 = 0.711, ns, and for interaction F1,18 = 3.627, *p* < 0.05.

### 2.4. [3H]Flunitrazepam Binding Autoradiography in WKY Female Rats

**Figure 3:**
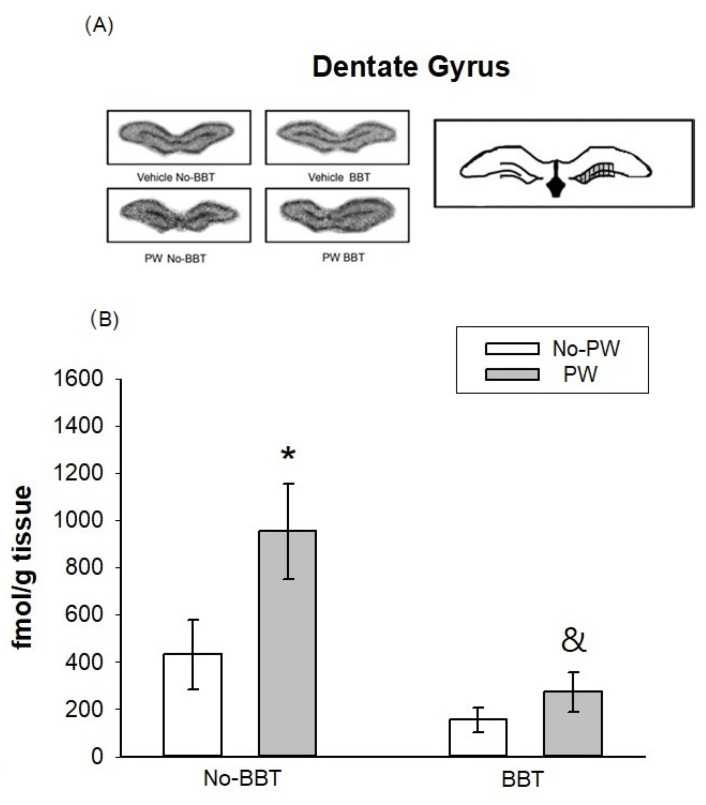
Representative photos of autoradiographic slides from the [3H]Flunitrazepam binding observed in the DG of the hippocampus (A), Effect of progesterone withdrawal (PW) on [3H]Flunitrazepam binding in the dentate gyrus of Wistar Kyoto rats ((**B**), n = 4–6 per group). No-BBT = not tested in the burying behavior test; BBT = tested in the burying behavior test. Each bar represents the mean value ± standard error of the mean. * *p* < 0.05 vs. No-PW; & *p* < 0.05 vs. No-BBT.

Figure 3A shows representative photos of autoradiographic slides from the [3H]Flunitrazepam binding observed in the DG of the hippocampus. In order to discard the effect of BBT exposure, a non-behaviorally tested group was included. Figure 3B shows that exposure to the BBT tended to reduce the [3H]Flunitrazepam binding in No-PW groups (*p* = 0.08). On the other hand, PW augmented [3H]Flunitrazepam binding in the DG only in rats that were not exposed to the BBT (*p* < 0.05). Effects due to behavioral test exposure in PW animals were observed (*p* < 0.005). Two-way ANOVA values were obtained for treatment F1,17 = 3.923, *p* = 0.05, behavioral test F1,17 = 8.745, *p* = 0.01, and interaction F1,17 = 1.557, ns.

**Figure 4:**
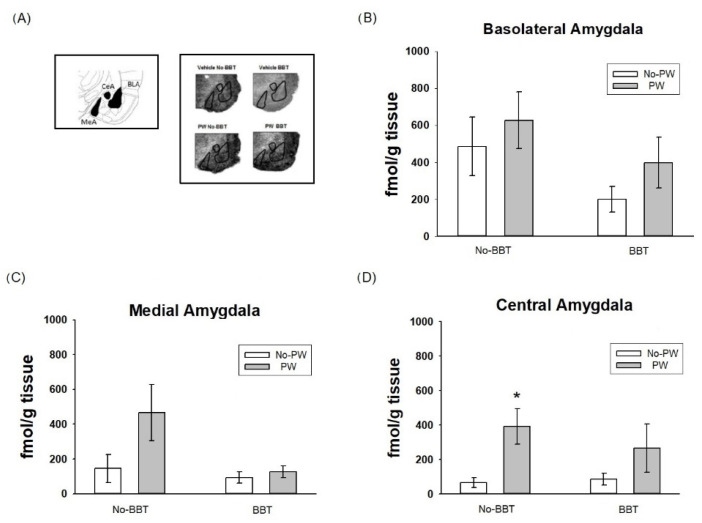
Representative images of autoradiographic slides from the [3H]Flunitrazepam binding observed in amygdala nuclei (**A**) Effect of progesterone withdrawal (PW) on [3H]Flunitrazepam binding in basolateral (**B**), medial (**C**), and central (**D**), amygdala Wistar Kyoto rats (n = 4–6 per group). No-BBT = not tested in the burying behavior test; BBT = tested in the burying behavior test. Each bar represents the mean value ± standard error of the mean. * *p* < 0.05 vs. No-PW.

Figure 4A shows representative images of autoradiographic slides from the [3H]Flunitrazepam binding observed in amygdala nuclei. No change was observed in the basolateral nucleus (Figure 4B) due to BBT exposure or the PW. Two-way ANOVA values were obtained for treatment (F1,14 = 1.358, ns), behavioral test exposure (F1,14 = 3.122, ns), and interaction (F1,14 = 0.386, ns).

No changes were observed in the medial amygdala (Figure 4C) in [3H]-Flunitrazepam binding due to PW or BBT exposure. Two-way ANOVA values were obtained for treatment (F1,14 = 2.178, ns), behavioral test exposure (F1,14 = 2.668, ns) and interaction (F1,14 = 1.443, ns). Lastly, in the central amygdala (Figure 4D), the PW increased the [3H]Flunitrazepam binding in No-BBT rats in comparison with the No-PW rats (*p* < 0.05), and no further changes were observed for BBT exposure, nor the combination of PW and BBT. Two-way ANOVA values were obtained for treatment (F1,14 = 6.69, *p* = 0.02), behavioral test exposure (F1,14 = 0.293, ns), and interaction (F1,14 = 0.560, ns).

### 2.5. Diazepam Effects on Anxiety-like Behavior in WKY Rats

**Figure 5:**
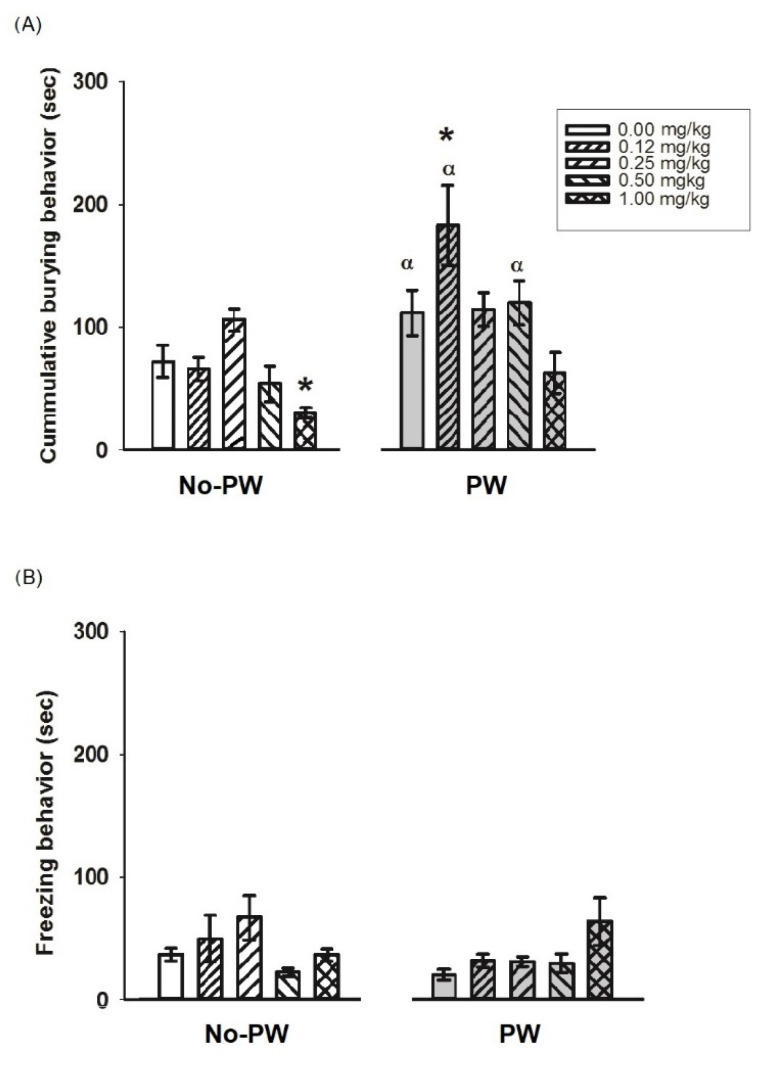
Effect of different doses of diazepam in Wistar Kyoto rats subjected to PW on cumulative burying behavior (**A**), and freezing behavior (**B**), registered in the burying behavior test (n = 5–7 per group). Data are presented as the mean ± standard error of the mean. * *p* < 0.05 vs. diazepam 0.00 mg/kg; α *p* < 0.05 vs. No-PW diazepam (different doses).

Diazepam at a dose of 1.0 mg/kg reduced the cumulative burying behavior in comparison with the control group (*p* < 0.05) (Figure 5A). PW exerted its characteristic anxiogenic-like behavior. Moreover, in animals subjected to PW, diazepam at a dose of 0.12 mg/kg induced an anxiogenic-like effect in PW animals treated with the diazepam vehicle (*p* < 0.005). PW and diazepam at a 0.50 mg/kg dose increased burying behavior compared with No-PW rats. Two-way ANOVA values were obtained for treatment (F1,60 = 25.14, *p* < 0.001), diazepam administration (F4,60 = 5.89, *p* < 0.001), and interaction (F4.60 = 3.10, *p* = 0.02). Regarding freezing behavior (Figure 5B), no changes were detected in response to diazepam treatment or PW. Two-way ANOVA values were obtained for treatment (F1,60 = 1.08, ns), diazepam administration (F4,60 = 2.07, ns) and interaction (F4,60 = 2.34, ns).

## 3. Discussion

The present data demonstrated that WKY rats showed anxiety-like behavior and higher corticosterone induced by PW alone. However, they showed a blunted HPA axis activity with the combination of PW and BBT exposure. Diazepam induced an anxiogenic-like behavior in response to PW effects that may parallel that observed in PMDD women.

Changes in the BDZ binding site in the GABAA receptor were observed in response to the PW, being more prominent in the DG of the hippocampus and central amygdala. In contrast, the BBT exposure per se did not modify the BDZ binding site in any of the brain areas examined. 

These findings suggest that PW can modulate the BDZ binding site of the GABAA receptor, but not for exposure to acute physical stress, such as BBT.

### 3.1. Progesterone Withdrawal Induced Anxiety-like Behaviors in Wistar Kyoto Rats

The present results showed that PW increased the anxiety-like behaviors evaluated in the two behavioral paradigms. We previously demonstrated that WKY rats were susceptible to PW by increasing anxiety-like behavior in the BBT [37]. The present study adds proof that PW induces anxiogenic-like effects in WKY rats in another animal model, the EPM. Our findings agree with previous results using other rat strains [37,52,53,54], where PW was used as a preclinical approximation to study premenstrual anxiety. Overall, our findings may parallel the anxiety observed in vulnerable women that presents as an adverse response to the abrupt drop of progesterone, which might play a relevant role in the etiopathology of PMDD [55,56].

PW but not BBT exposure increased corticosterone levels, suggesting that the WKY strain is more sensitive to hormonal than physical challenges. Therefore, the HPA axis stimulation in WKY rats seems more reactive to physiologic stressors (like PW). This proposal is in line with previous human evidence, as reports have shown increased plasma cortisol in response to a physiologic challenge by CRH administration in PMDD women, compared with controls [57]. Furthermore, physical stress through exercise failed to induce an increase in cortisol in women with PMS [10]. Notably, the combination of PW and BBT failed to increase corticosterone levels, suggesting a blunted HPA axis response. Interestingly, this parallels previous findings in women with premenstrual mood disorders, as reports have found attenuated HPA axis activity in response to acute stress [19,58,59] that might be related to an impaired stress-coping mechanism.

### 3.2. [3H]Flunitrazepam Receptor Binding in WKY Rats Subjected to the Burying Behavioral Test and/or Progesterone Withdrawal Challenge

Differences were found in [3H]Flunitrazepam binding in response to the type of stressor depending on the rat brain area studied. Thus, in response only to PW, an increase in [3H]Flunitrazepam binding sites was observed in the DG and central amygdala. In support of this finding, Dayas et al. (2001) [47] observed that the central amygdala was activated by a systemic stressor (hemorrhage or immune challenge). Concerning the DG, to our knowledge, no previous report has addressed the impact of different types of stressors in rats. The brain areas analyzed for [3H]Flunitrazepam binding are involved in anxiety regulation [60,61], and although the BBT involves exposure to an aversive electrified prod [62], per se it did not induce changes in [3H]Flunitrazepam binding in WKY rats in any brain area evaluated. 

Stressors can be classified according to their nature into “systemic” or “processive” (physical) stressors [63]. Based on this proposal, PW could be considered a systemic stressor, while the BBT could represent a physical stressor. Thus, it is possible that BBT did not alter BDZ binding in the GABAA receptor due to a higher sensitivity to physiological, rather than physical, stressors. Although this idea remains to be explained, differences in sensitivity to a variety of challenges might be related to stress susceptibility, as WKY rats are considered a stress-vulnerable strain and might resemble a chronic stress condition reflected by hyperactivation of the HPA axis [39,40,64]. 

In this sense, it has been reported that under a chronic stress condition, the composition of GABAA receptor subunits and their functionality may change to facilitate sensitivity to different stimuli, even to GABAA modulators (reviewed in [26,65]) and its withdrawal [25,66]. Therefore, it might be the case that the higher binding to [3H]flunitrazepam after the PW challenge observed in DG and the central amygdala was related to a particular conformation of GABAA receptor subunits. Future studies will aid in confirming this hypothesis.

### 3.3. Behavioral Effect of Diazepam on Anxiety-like Behaviors Induced by PW

Highly anxious rodents and rodents exposed to chronic stress have significantly decreased GABAA/BDZ receptor-mediated chloride ion flux, lower GABAA/BDZ receptor expression, and reduced sensitivity to neurosteroids and benzodiazepines [67,68,69,70,71,72]. Here, we observed that WKY rats were not responsive to the anxiolytic effects of low doses of diazepam, only to a dose of 1 mg/kg, which decreased active anxiety-like behavior (in the absence of PW). Interestingly, this behavioral pattern was disturbed by PW, as diazepam induced anxious-like behavior at a dose of 0.125 mg/kg. This anxiogenic-like effect of diazepam was in line with previous findings in women with premenstrual mood disorders, as an abnormal response was observed with other positive modulators of the GABAA receptors, such as ALLO, BDZs, or alcohol [32,73,74]. In addition, it has been proposed that chronic stress may facilitate the paradoxical effects of GABAA modulators along with steroid fluctuations (for review, [75]). In line with this proposal, WKY rats showed increased HPA axis activation [40,64], which may resemble a chronic stress-like condition and could explain the paradoxical effect of diazepam observed in the PW WKY rats. 

Conversely, we did not observe changes in freezing behavior, which could be related to the manipulations performed, as the rats were manipulated for at least five days before the BBT (due to the injection schedule of the PW). In this sense, it is known that previous handling could attenuate the behavioral stress response in WKY rats [76] Therefore, it is possible that handling could have masked the potential differences in passive anxiety-like behavior in our experimental conditions.

Collectively, the results suggest that the WKY rat strain may be an experimental subject that meets some PMDD traits regarding the GABAergic system and HPA axis activity. Thus, WKY rats could represent an alternative animal model to study potential treatments for the anxiety symptoms of PMDD patients. The limitations of the present study included that we did not evaluate the subunit composition of GABAARs in brain areas of WKY rats or the binding of other subunits of this receptor; future studies could provide more evidence on the GABAAR function in this strain. Another limitation was that we did not test the GABAAR binding and diazepam effects in rats tested in the EPM. Therefore, future experiments will strengthen the convergence validity of the present findings.

## 4. Materials and Methods

### 4.1. Animals

A total of n = 118 adult (3 month old) female Wistar Kyoto (WKY) rats from our breeding facilities (Cinvestav, Mexico City, Mexico) were used for all experimental series. Animals weighing 250–300 g were housed (5–6 per cage) in polycarbonate cages in a room with a controlled temperature (22 to 24 °C) under a 12:12 h light–dark cycle (lights off at 10:00 a.m.) with continuous access to water and food. All procedures were approved by the local Ethics Committee on Animal Experimentation and followed the official Mexican norm for animal care (NOM-062-ZOO-1999). All efforts to reduce the suffering and number of animals were applied. 

### 4.2. Ovariectomy

Animals were ovariectomized (OVX) under anesthesia with 2% tribromoethanol (10 mL/kg i.p.), and a ventral incision was performed to expose and remove the ovaries. Before induction of anesthesia, animals were given 1 mg/kg of meloxicam *p.o.* After suturing muscles and skin, topic antiseptic was applied directly to the wound, and an antibiotic was given at a dose of 15 mg/rat *i.m.* Once surgery was completed, rats were returned to their home cage and allowed a 3 week recovery period [37,77]. Once recovered, rats were randomly assigned to different groups and treatments. 

### 4.3. Progesterone Withdrawal (PW)

To induce PW, OVX rats were injected subcutaneously with 2.0 mg/kg, s.c. progesterone (Sigma-Aldrich, Toluca, Mexico) or vehicle (corn oil) once a day for five consecutive days. Twenty-four hours after the last injection (of progesterone or vehicle), rats were subjected to the locomotor activity test, the burying behavior test, or the elevated plus maze. Dose and time point for PW were based on previous studies [37,53,54] showing that PW induced anxiety-like behaviors in the BBT 24 h after the last progesterone injection, compared with No-PW vehicle-treated animals. All treatments were administered between 9 and 10 a.m. Progesterone was dissolved in corn oil and injected in a 1 mL/kg final volume.

### 4.4. Behavioral Models

All behavioral assessments were performed between 9 and 10 a.m. in a quiet isolated room, lit with a red bulb. After each test, all apparatuses were thoroughly cleaned with a solution made of ethanol and Extran^®^. During the behavioral task, the experimenter remained at the room’s entrance (2 m away from the apparatus) to assure optimal conditions throughout the behavioral assessment. Data collection was always performed by an individual blind to the treatments.

#### 4.4.1. Locomotor Activity Test

The animals were tested in the locomotor activity test to discard the potential influence of treatments on general locomotor activity. The apparatus consisted of an opaque Plexiglas^®^ cage (40 × 30 × 20 cm) with the cage floor divided into 12 equal squares. The animals were placed in a corner of the cage, and an observer registered the number of squares crossed in a 5 min session. The crossing was considered as occurring when the animal had three-quarters of its body inside the quadrant [77]. Once locomotor assessment was completed, there was a 15 min intertrial interval (ITI) before the animals underwent the next behavioral test.

#### 4.4.2. Elevated Plus-Maze Test

The elevated plus maze (EPM) consisted of a cross-shaped apparatus that was 50 cm above the floor and displayed two different zones: one was a potentially aversive zone (open arms), and the other was considered a safe zone (closed arms) [78]. The four arms of the maze were 50 cm (length) × 10 cm (width). Two opposing arms had plastic walls of 40 cm (height, closed arms), and the other two arms lacked walls (open arms). The test commenced when the animal was placed in the maze’s center, facing a closed arm. The session was video recorded (Canon PowerShot sx500 IS video-camera) for 10 min. The behavioral parameters registered were: (a) the cumulative time spent in the open arms, expressed as % of the time; (b) the cumulative time in the closed arms, expressed as % of the time; and (c) the number of total arm crosses. Thus, the more time the animal spent in the open arms, the less anxiety-like behavior; conversely, the more time spent in the closed arms, the higher the anxiety index was interpreted [78]. The total number of crosses was related to the general locomotor activity of the rat [79].

#### 4.4.3. Defensive Burying Behavior Test (BBT)

This animal model allowed for the recording of different behaviors and a better understanding of the rat’s behavioral response to an aversive stimulus [41]. Overall, the behaviors displayed in this paradigm were divided into active (i.e., burying time) or passive (i.e., freezing) behaviors [41,50].

The BBT was performed as previously described [80]. The experimental acrylic cage (27 × 16 × 23 cm) contained an electrified prod (7 cm long) that emerged 2 cm above the bedding material (fine sawdust), from one of the walls. The electrified prod was considered an acute physical stressor. Every time the animal touched the prod, an electric shock of 0.3 mA was supplied by a constant current shocker (LaFayette Instruments Co., model 5806, Lafayette, IN, USA). Once the animal was placed in the cage, the behavioral test commenced, and it was video recorded (Canon PowerShot sx500 IS video-camera) for 10 min. Once the animal received the electric shock, it typically drove its attention to the prod, recognizing it as the aversive stimulus. Then, the animal sprayed and pushed a pile of bedding material ahead with rapid alternating movements of its forepaws. The parameters registered were the cumulative burying behavior (time in seconds that animals spent burying the prod) and freezing behavior (time in seconds without any movement after receiving a shock) [50]. Both parameters were considered an index of anxiety-like behavior: cumulative burying behavior indicated an active coping, while freezing indicated a passive anxiety displaying strategy [37,41,50]. In all cases, 1 h after receiving vehicle or progesterone injection, the animals were habituated to the experimental cage without the prod for 10 min per day for three consecutive days before behavioral testing. 

### 4.5. Determination of Corticosterone Levels

As an index of HPA axis activation, corticosterone (CORT) levels were determined from plasma samples using the corticosterone immunoassay kit (Assay Designs, Inc., Ann Arbor, MI, USA according to manufacturer instructions. The microplate was read at 405 nm in an ELISA reader (Bio-Tek, Winooski, VT, USA). Inter- and intra-assay variabilities were 7.8 and 6.6%, respectively.

### 4.6. Evaluation of BDZ Binding Site in the GABAA Receptor Using [3H]-Flunitrazepam Autoradiography

Quantitative autoradiography of [3H]flunitrazepam binding in the dentate gyrus of the hippocampus and amygdala basolateral, central and medial was performed. These brain areas were selected due to their involvement in the HPA axis regulation through the BDZ binding site of the GABAA receptors [59] and their essential role in regulating anxiety-like behavior [60]. Brains were quickly removed, flash-frozen in isopentane over dry ice, individually wrapped in aluminum foil and stored at −70 °C. Frozen coronal sections (20 µm thick) were cut in a cryostat, thaw-mounted on gelatin-coated slides, and maintained at −70 °C until processed. Following Paxinos and Watson’s rat atlas [61], adjacent coronal sections (six per animal) were cut from −2.8 to −3.3 mm from the bregma.

Autoradiography of BDZ’s specific binding site of the GABAA receptor was performed as described by [62]. On the incubation day, the brain sections were pre-washed in Tris-HCl buffer (170 mM; pH 7.4) for 30 min at 25 °C. Then, the BDZ binding site was labeled with [3H]flunitrazepam (2 nM; 82.5 Ci/mM Amersham, Arlington Heights, IL, USA) and incubated for 45 min at 4 °C. After incubation, sections were washed twice for 1 min each time in a fresh buffer and rinsed in distilled water (2 s) at 4 °C. The sections were then quickly dried under a gentle stream of cold air. Non-specific binding was measured in the presence of Clonazepam (1 µM). The slides were arrayed in X-ray cassettes with tritium standards (Amersham) and exposed to tritium-sensitive film (Amersham Ultrafilm) for three weeks at room temperature. The films were developed using standard Kodak GBX and a fixer at room temperature. 

Optical density (OD) was converted into a radioactivity value using an imaging system for autoradiography densitometry. Tissue sections were accompanied by a series of 12 precalibrated standards (Amersham, Arlington Heights, IL, USA) placed on each autoradiogram. Each autoradiograph was transformed into a digital display through a video camera. The analysis was performed in an automated computer and densitometer reconstruction of digitized film autoradiographic images using JAVA (Jandel Video Analysis Software). Brain regions, such as the DG of the hippocampus and the medial, central, and basolateral amygdala nuclei, were analyzed in the same slides localized by reference to the rat atlas [81]. OD readings were recorded and averaged from at least five brain sections. Then, the OD readings from the standards were used to determine the tissue radioactivity values. Quantification was made using the reference curve built from commercial plastic-impregnated radioactive standards (Amersham, Arlington Heights, IL, USA). The gray value of each pixel in an autoradiographic image was converted into a corresponding radioactivity concentration by interpolation in the calibration curve. Specific binding was estimated by subtracting the non-specific binding in all experiments.

### 4.7. Effects of Diazepam on Anxiety-like Behavior Induced by PW

Diazepam was dissolved in propylene glycol 40% and administered intraperitoneally (i.p.) 30 min before the behavioral test [48] and a dose–response curve was constructed at doses 0.0, 0.12, 0.25, 0.5 and 1.0 mg/kg. Latency and doses were taken from previous studies [48,77].

### 4.8. Experimental Design

*Experiment 1: Effect of PW challenge on anxiety-like behavior and corticosterone levels.*

**Figure 6:**
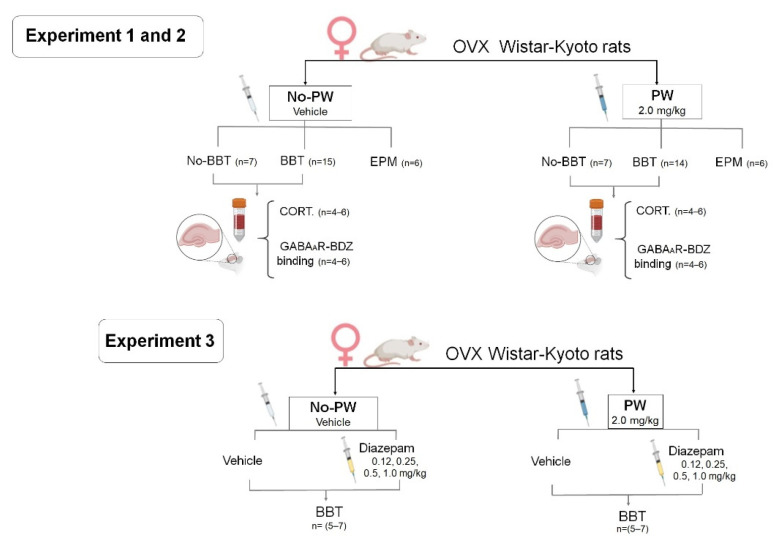
Flowchart of experimental design.

Overall, the experiment lasted five weeks from rats’ arrival to euthanasia. Animals were given a one-week acclimation period before ovariectomy. All animals had three weeks post-OVX before experimental assignment. Using G power 3.1 statistical software [82] and based on previous means from anxiety-vehicle-treated rats and PW animals [54], a coefficient of size effect was obtained. Thus, G power yielded a minimum of n = 14 animals per group for the BBT, and n = 6 animals per group for EPM. Animals were split into two groups: the first was assigned to the EPM assessment, and the other to the BBT. Once divided, two independent groups per behavioral test were conformed: (1) No-PW (vehicle-treated group; BBT n = 15, EPM n = 6), and (2) rats subjected to the PW schedule (PW; BBT n = 14, EPM n = 6) as described above (see Section 2.3). Twenty-four hours after the last administration of progesterone, animals were subjected to the BBT or the EPM. Only those animals that were evaluated in the BBT were tested in the locomotor activity test 15 min prior. After 60 min of the BBT, animals were euthanized by decapitation [37], and the trunk blood was collected and centrifuged at 30,000 rpm for 30 min. An OVX No-PW control group not exposed to the locomotor activity test nor BBT was included to obtain a basal measure of corticosterone (n = 4–6 per group) to discard the effects of acute stress due to behavioral test exposure. Serum samples remain stored at −80 °C until processed. The schematic representation of the experimental timeline is shown in Figure 6.

*Experiment 2: Effect of PW challenge on [3H]-Flunitrazepam autoradiography as an index of BDZ specific binding site in GABAA receptors*.

The brains of animals from Experiment 1 subjected to the BBT (No-PW and PW, n = 4–6 per group) were extracted after decapitation and used for autoradiography examination of the amygdala and DG of the hippocampus since these areas are strongly involved in the regulation of anxiety behavior [83,84,85]. The same control group from Experiment 1, not exposed to BBT, was included to obtain a basal measure of [3H]flunitrazepam autoradiography.

*Experiment 3: Response to different doses of Diazepam in WKY rats subjected to PW.*

Independent groups of rats were conformed (No-PW and PW, n = 5–7 per group). All received a single administration (i.p.) of a different dose of diazepam as follows: 0.0, 0.12, 0.25, 0.5 and 1 mg/kg. After 30 min of latency, all animals were tested in the locomotor activity test and the BBT [48,77].

### 4.9. Statistical Analysis

Results were shown as the mean ± standard error of the mean (SEM). Behavioral data were analyzed through the Student’s t-test comparing No-PW vs. PW groups. A two-way ANOVA test was performed considering behavioral test exposure (No-BBT or BBT) and treatment (No-PW or PW) as factors for the autoradiography study and corticosterone levels determination. Additionally, a two-way ANOVA test was applied for the diazepam dose–response curve, considering the factor treatment (No-PW vs. PW) and diazepam (vehicle vs diazepam doses). The Holm–Sidak *post hoc* test followed the analysis. When necessary, specific comparisons were made through the t-test. Values of *p* < 0.05 were considered statistically significant. 

## 5. Conclusions

Of relevance to the homological validity criterion for animal models (Belzung and Lemoine, 2011), this study observed that WKY rats had a blunted HPA axis response in corticosterone levels, altered GABAA receptor functionality, and higher anxiety-like behavior induced by PW that may resemble characteristics of PMDD, that requires further exploration.

## Figures and Tables

**Table 1:** Effect of progesterone withdrawal and different doses of diazepam on the total number of squares crossed in the locomotor activity test in Wistar Kyoto rats.

Treatment	Squares Crossed (n)
No-PW	31.6 ± 1.8
PW	34.8 ± 1.7
DZ 0.0	20.2 ± 0.7
DZ 0.125	19.6 ± 1.6
DZ 0.25	21.8 ± 2.4
DZ 0.50	22.0 ± 2.7
DZ 1.0	18.6 ± 1.4
PW + DZ 0.0	19.4 ± 0.8
PW + DZ 0.125	20.4 ± 1.3
PW + DZ 0.25	21.4 ± 1.5
PW + DZ 0.50	21.6 ± 1.3

Data are expressed as mean ± standard error of the mean. No-PW = no progesterone withdrawal (vehicle-treated rats); PW = progesterone withdrawal; DZ = diazepam.

## Data Availability

The data presented in this study are available on request from the corresponding author.
